# Annual Report of Brig.-General George M. Sternberg

**Published:** 1899-01

**Authors:** 


### ANNUAL REPORT OF BRIG.-GENERAL GEORGE M. STERNBERG.

Surgeon-General U. S. Army.

[*Abstract.*]

### HEALTH OF THE TROOPS.

In my opinion the reduction of the age limit from twenty-one to eighteen years and the haste with which the volunteer regiments were organised and mustered into the service were responsible for much of the sickness which was reported in the early days of their camp life. All military experience shows that the young men under twenty-one years break down readily under the strain of war service, and every regiment had many of these youths in its ranks. Medical examiners were appointed to testify to the physical qualifications of each man before acceptance, but, notwithstanding this, which at the time was characterised in the press as a very rigorous procedure, so many men were afterward found on the sick lists of the camps unfit for service, from causes existing prior to enlistment, that special arrangements had to be made for their discharge.

Soon after the newly-raised levies were aggregated in large camps sickness began to increase progressively from causes that were so general in their operation that scarcely a regiment escaped from their harmful influences. These causes may largely be referred to ignorance on the part of the officers of the principles of camp sanitation, and of their duties and responsibilities as regards the welfare of the enlisted men in their commands. Medical officers, as a rule, were also without experience in the sanitation of camps and the prevention of disease among troops. The few who knew what should be done were insufficient to control the sanitary situation in the large aggregation of men hastily gathered together. Officers and men in these camps were rife for war, and drill, parades, practice marches and military camp duties occupied the whole of their time and energies. Considerations of domestic economy and sanitation in the companies and regiment were not given proper attention, and men who were being taught to meet the enemy in battle succumbed to the hardships and unsanitary condition of life in their camps of instruction.

The sites of certain of the camps have been instanced in the newspapers as the cause of the sickness which was developed in them; but a review of the whole situation shows that it was not the site, but the manner of its occupation, which must be held responsible for the general spread of disease among the troops. On April 25, 1898, foreseeing the likelihood of unsanitary conditions in the camps of our newly-raised troops, and with the view of preventing them I issued Circular No. 1 from this office, impressing upon medical officers their responsibility in sanitary matters and the necessity for a strict sanitary police, particularly in the care of the sinks and in the preservation of the camp area from contamination. But the density of the military population on the area of these contracted camps prevented the possibility of good sanitary condition. Camps of this character may be occupied for a week or two at a time without serious results, as in the case of National Guardsmen out for ten days' field practice during the summer, but their continued occupation will inevitably result in the breaking down of the command by diarrhea, dysentery and typhoid fever.

### UNSANITARY CONDITIONS REMEDIED.

Practically nothing was done to make the men comfortable or to remedy the unsanitary conditions until these were brought to the attention of the Secretary of War by inspectors sent out by special orders from the War Department. Then the camps held for so long were abandoned, but not before the manifestations of typhoid infection were rife in them. New sites were carefully selected, regimental camps were expanded, company tentage increased and board flooring provided. Then, for the first time, the troops went into camps suitable for continued occupation.

One prominent cause of the increase of sickness in the early camps has been commented upon by only a few of our medical officers. These cite the prevalence of drunkenness and of disease, due to the facilities and temptations afforded by the proximity of cities to the larger camps. They hold that if the systems of the men had not been weakened by dissipation they would not have succumbed so readily to the other influence which affected them.

Malarial fevers added to the sick lists of camps in Florida and of Southern regiments in the camps of Georgia and Virginia.

It was, however, typhoid fever which broke down the strengths of the commands generally, the outbreak becoming distinctly manifest in July. Sporadic cases appeared in most of the regiments in May and June, these cases having been brought in many instances from the state camps. In fact some regiments, as the 15th Minnesota, suffered more from this disease at their state rendezvous than any of the regiments in the large Federal camps. A few of the regimental commands in the latter may be said to have escaped visitation. The sanitary conditions affecting the commands in the various camps have been studied in connection with the prevalence of typhoid fever among the men by a board of medical officers, consisting of Majors Reed, Vaughan and Shakespeare, but the results of the investigation of this board have not as yet been reported in full. It appears to me, however, from a general review of the sanitary reports already filed, that the prevalence of the disease was proportioned to the unsanitary camp conditions which I have referred to. My circular No. 1, already cited, was intended to bring the danger from this fever to the notice of medical officers, with the view of obviating it. The probability of its communication to soldiers in camps through the agency of flies was pointed out as a reason for insisting on a sanitary police of the strictest character.

It is well known to the medical profession that this fever is propagated by a contaminated water supply, and it is now recognised that the great prevalence of this disease in an aggravated form in the camps of the Civil War was due to the use of surface and shallow well waters infected by typhoid excreta. To prevent transmission by the water supply I recommended the use of boiled and filtered water when a pure spring supply could not be obtained, and to enable an efficient filtration of suspected waters to be made, field filters of approved construction were issued on recommendation by the Quartermaster's Department.

### CARE OF THE SICK AND WOUNDED.

As soon as the regiments were organised into brigades and divisions, preparatory to active service, it became the duty of each chief surgeon of an army corps to see that the medical department of his command was organised to meet the casualties of battle. The object of the concentration of the troops was to accustom the regiments to operations in which they constituted the units of a higher organisation.

Circular No. 2, from this office, dated May 18, 1898, in specifying the duties of the various medical officers of the army corps, indicated the character of the organisation to be adopted. The seriously sick were to be treated in division field hospitals (unless their transfer to a general hospital was advisable), under the care of the most experienced physicians and able surgeons on duty with each division. Medical officers left on duty with their regiments were to exercise sanitary supervision over the well men and to determine whether a soldier reporting himself sick should be sent to hospital or remain as a trivial case under treatment in quarters. This consolidation of the medical force by divisions, implying, as it did, the breaking up of the regimental hospitals, met with a strong opposition from regimental medical officers, particularly from those who were not detailed for special service at the division hospitals. Regimental commanders also were in many instances opposed to it, forgetful that the object of the medical department, as of the line, was to get into training for field service. Similar objections were raised in 1862 and 1863 to the establishment of the division hospitals, but the Civil War instead lasted long enough to demonstrate the superiority of this system.

### THE FIFTH ARMY CORPS.

Long before the Fifth Army Corps embarked for Cuba its field hospitals were in condition for efficient service. Subsequent events, however, rendered valueless these preparations of the Medical Department. When the command embarked on the transport vessels, the baggage wagons and mules were left behind. The ambulance trains of all the divisions, with a large part of the outfit of each of the hospitals, were also left behind. Three ambulance wagons were taken apart and stored on one of the vessels. These did excellent service at San Juan and El Caney. Ten of the ambulances of the Third or Reserve Divisional Hospital were subsequently shipped to Cuba, where they arrived July 2d and were of value in moving the sick and wounded to the hospital at Siboney and to the hospital ships and transports.

Of the property and supplies carried to Cuba, a portion was not available for service at the time it was most needed, to-wit, on July 1st, 2d and 3d, when the wounded from El Caney and San Juan were coming from the front for care and treatment. This was because, in general, no opportunity was afforded to land the medical property. Earnest efforts were made by medical officers to have supplies at the front with the troops. Some having succeeded in getting their medicine chests and other articles of medical property ashore, had these carried forward on litters by hospital corps men to the camps near

Sevilla, while others turned their private mounts into packhorses for this purpose. During and after the battles at El Caney and San Juan there was an insufficiency of tents, cots, bedding and medicines, due to the causes stated, but all the hospitals were well equipped for surgical work.

After the capitulation of Santiago the troops at the front broke down rapidly under the fatigues they had undergone and the malarial influences to which they were exposed, but by this time an ample supply of tents, furniture, bedding, clothing and medical stores had reached Siboney, together with a corps of trained nurses and a force of surgeons, those sent to duty at the yellow fever hospital being immune to that disease. Meanwhile, to relieve the pressure on the field hospitals, such convalescents and sick as could bear the journey home were sent to the United States on transport vessels. This was an emergency measure, to relieve the hospitals at Siboney and permit of the transfer to them of the men who were sick in regimental camps.

The transfer of troops from Santiago to Montauk Point, New York, was also an emergency measure, and the great responsibility of excluding yellow fever infection from every transport rested on the medical officers who had charge of the embarkation. Had they failed in this duty, the effect would have been disastrous during the voyage to the men confined on shipboard, and the risk of importing the disease into this country would have been greatly increased.

### THE CAMP AT MONTAUK.

In view of the necessity for the return of the troops of the Fifth Army Corps from Santiago, Cuba, preparations were made for encamping them at Montauk Point, Long Island. These included the establishment of temporary tent hospitals, not only for the treatment of the large number of sick brought by each command from Cuba, but for the isolation and treatment of these from transports lying under the suspicion of yellow fever infection.

The difficulties in the way of administering the affairs of the detention hospital were very great owing to the rapidity with which the transports followed each other in their arrival. As many as four reached the Point on some days from August 12th to August 31st, most of them bringing sick requiring detention for medical observation; but the sick men were as well cared for and as comfortable in their cots here as afterward, when transferred to the general hospital at Montauk Point. There was an excellent steam disinfecting plant on the grounds, with a formaldehyde chamber attached. The laundry work was done at a steam laundry near the hospital.

The temporary hospital, which was locally known as the general hospital, Montauk Point, consisted of tent pavilions, containing 1,672 cots. Its personnel consisted of 40 medical men, 8 stewards, 10 acting stewards, 130 privates of the hospital corps, 15 cooks and 50 male nurses, and an average of 200 female nurses, one-half of whom were Sisters of Charity. Supplies of all kinds were amply provided.

It is needless to refer at this time to the complaints of starvation which appeared almost daily in the newspapers during the occupation of Camp Wikoff, for it is now generally understood that the weakness, prostration, anemia and emaciation of so many of the troops were the results of malarial, typhoid and yellow fevers, from which the Army suffered as a consequence of its exposure to the climatic influences and local infections of Santiago and its neighborhood pending and subsequent to the surrender of the city.

TROOPS IN THE HOME CAMPS.

The method of hospital organisation in the home camps was practically the same and there was much similarity in the conditions affecting them and correspondingly in their history. Regiments reported in but few instances with the material and supplies for their medical care, but they brought sick men with them, and these required immediate care. Provision had to be made for division hospitals, in view of the future field service, and for regimental hospitals, in view of the immediate necessity. The difficulties in the way of the contemporaneous accomplishment of these two objects were great, and they were greatly augmented by the inexperience of a majority of the regimental medical officers, and of many of the chief surgeons, which prevented them from seeing beyond the immediate necessity. The sick had to be cared for, and to this end medicines and other things had to be procured.

Relief societies offered assistance, and this was eagerly accepted by many of these medical officers, not alone for delicacies or luxuries not otherwise provided for, but for "supply-table" articles, which could have been had from the medical purveyors in their camps, or by telegraphic requisition on the Surgeon-General. It was easier to accept what was so freely offered than to learn how to obtain the articles from the proper source. To explain their prompt acceptance of this assistance, these officers referred to the red tape of the War Department methods, and the insinuation that the said methods were beyond the comprehension of the ordinary intellect was accepted by the sensational press as an explanation in full.

Meanwhile chief surgeons of corps and divisions began the organisation and equipment of their field division hospitals and ambulance companies, but they were met at the outset by the apparent impossibility of securing men for service as cooks, nurses, litter bearers, ambulance drivers, teamsters, etc. The Hospital Corps of the Regular Army could not supply these men, because recruiting for this corps progressed slowly. The popular tendency to volunteer led men away from the regular recruiting offices. When transfers from the volunteer regiments to the regular Hospital Corps were authorised, the men did not care to leave their local connections for service in the army at large as regular soldiers. The transfers, so much desired by the Medical Department to enable it to complete its organisation, were not regarded favorably by line officers, for, although every line officer will probably acknowledge as a general principle that only the most intelligent and capable men should be employed to care for the sick and wounded, he is not likely to act on this general principle when it is a question of withdrawing for such service the most intelligent and capable men of his own company or regiment.

### THE DIVISION HOSPITALS.

The division hospitals of the Army Corps were usually established in the immediate neighborhood of the regimental camps of the divisions. The pavilions were arranged in various ways, according to the configuration of the area available as a site; but in general there was a tendency to crowd the area. Surgeons in charge recognised that a tent should not be occupied by more than six patients, but sometimes this number was exceeded temporarily while waiting an increase of tentage. As a rule the hospitals were kept in campaigning condition, that is, the tents were neither framed nor floored, until the increased prevalence of typhoid fever attracted attention to their crowded condition, when the object of their existence became suddenly changed from a school for field service to a hospital for the treatment of a local outbreak of disease.

Special diet kitchens, under the management of capable individuals, were opened at most of the hospitals. Money for this purpose was sent to them by me from funds contributed and placed at my disposal. Money was also sent directly by individuals and representatives of aid societies, and the Red Cross committees supplied quantities of ice and milk, chicken, eggs, lemons, etc., pajamas, night shirts and other articles of hospital clothing, were also provided by the Red Cross and other societies. Subsequently the order authorising the commutation of the sick soldiers' ration at 60 cents rendered these hospitals wholly independent of outside assistance.

### MEDICAL STATISTICS OF THE WAR.

The work of gathering up the records of sickness of the various commands in service during the war has been one of great difficulty. Volunteer medical officers were ignorant of the methods of keeping their records, and many failed to appreciate the importance of what was frequently regarded as "mere paper work" which had no practical bearing on the welfare of the men. Nevertheless, their work in this regard must be considered as satisfactory when compared with that of the volunteer medical officers of the War of the Rebellion.

My report represents tabulations compiled from monthly reports of sick and wounded received from May to September, inclusive, and representing a strength present of 167,168 men. These give full particulars of 1,715 deaths, of which number 640 were occasioned by typhoid fever, 97 by malarial fevers and 363 by diarrhea and dysentery. The death rates for May and June, .46 and .70 were not in excess of those of the Army in time of peace. In July the rate became somewhat higher than that of most well-cared-for cities, 2.15 for the month, or the equivalent of an annual rate of 25.80 per thousand living. In August it became excessive, 4.08 for the month, equal to an annual rate of 48.96 per thousand. In September the influence of the energetic measures taken in July and August to improve the health of the army, become manifest in the falling of the death-rate to 2.45, or the equivalent of an annual rate of 29.40. The same progression to an acme in August, with a sudden fall in September, is seen in the various ratios given under the specific titles, typhoid fever, malaria fever and diarrheal diseases. This is exceedingly gratifying, and must be credited, as stated, to the sanitary measures adopted, for our experience in the Civil War demonstrates that in the absence of these measures the high ratio of August would have been continued for many months to come.

I submit also tables of absolute numbers and the ratio by which the incidence of sickness and mortality of the regular and volunteer troops may be contrasted. From these it will be seen that the exposures of the regular troops during the Santiago campaign gave them from June to September a higher death-rate than the volunteers, and that the rate of the latter during August, the month of maximum mortality, was 3.62, as compared with 5.83 among the regular troops.

### VOLUNTEER RELIEF WORK.

My guiding principle throughout the war has been that relief, when needed, should be promptly accepted, without reference to the source from which it came. The relief afforded by the National Red Cross at Siboney was promptly accepted by the surgeons on the spot, but it is evident that it was entirely inadequate to meet the emergency. This association has had full authority to send agents and supplies to all of our camps since June 9, 1898, and it has contributed supplies of various kinds in almost liberal manner for the use of our field hospitals. Other organisations which have rendered very valuable services are the National Relief Commission, having its headquarters in Philadelphia and the Massachusetts Volunteer Aid Association, with headquarters in Boston. Both of these organisations fitted out hospital ships, which were placed at my service for the transportation of our sick from Porto Rico, and I take pleasure in testifying to the valuable services rendered by the yacht May, of the National Relief Commission, and the hospital ship Bay State, of the Massachusetts Volunteer Aid Association.

